# Use of self-expandable metallic stents for endoscopic biliary decompression decreases stent complications in pancreatic cancer patients receiving chemotherapy

**DOI:** 10.1007/s00464-021-08327-y

**Published:** 2021-02-03

**Authors:** Sini Vehviläinen, Hanna Seppänen, Anna Nurmi, Caj Haglund, Harri Mustonen, Marianne Udd, Leena Kylänpää

**Affiliations:** 1 Department of Gastrointestinal Surgery, Helsinki University Hospital, Helsinki, Finland; 2 Translational Cancer Medicine Research Program, University of Helsinki, Helsinki, Finland; 3 Department of Surgery, Meilahti Hospital, University of Helsinki, Haartmaninkatu 4, PO Box 340, HUS, 00029 Helsinki, Finland

**Keywords:** Pancreatic cancer, Endoscopic biliary decompression, SEMS, Chemotherapy, Cholangitis

## Abstract

**Background:**

Both plastic stents and self-expandable metallic stents (SEMSes) are used for endoscopic biliary decompression (BD) among patients with pancreatic cancer (PAC). Cholangitis or stent occlusion often interrupts or ends chemotherapy. We investigated cholangitis, stent occlusion, and chemotherapy interruption rates for SEMSes and plastic stents among patients receiving chemotherapy for PAC.

**Materials and methods:**

We retrospectively analyzed data for 293 PAC patients who received a biliary stent at Helsinki University Hospital during 2000–2017. Patients received chemotherapy as palliative treatment (PT: *n* = 187) or neoadjuvant treatment (NAT: *n* = 106). Among participants, 229 had a plastic stent (PT: *n* = 138, NAT: *n* = 91) and 64 had a SEMS (PT: *n* = 49, NAT: *n* = 15).

**Results:**

Overall, 15.6% (*n* = 10) of patients with SEMSes (PT: 20.4%, *n* = 10, NAT: 0%) and 53.0% (*n* = 121) of patients with plastic stents (PT: 69.3%, *n* = 95, NAT: 28.5%, *n* = 26) experienced one or more stent complications (*p* < 0.001). Cholangitis developed in 6.3% (*n* = 8) of PT patients with SEMSes. No patients with SEMSes receiving NAT (*n* = 15) experienced cholangitis. However, 31.9% (PT: 42.8%, *n* = 59, *p* = 0.001; NAT: 15.4%, *n* = 14, *p* = 0.211) of patients with plastic stents developed cholangitis. Among all patients receiving NAT or PT, cholangitis interrupted chemotherapy 6 times (9.4%) in SEMS patients and 61 times (26.6%) in plastic stent patients (*p* = 0.004). Stent occlusion without cholangitis interrupted NAT or PT 2 times (2.1%) in SEMS patients and 31 times (13.5%) in plastic stent patients (*p* = 0.023).

**Conclusions:**

SEMS is recommended for BD among patients with PAC receiving chemotherapy. Among both PT and NAT patients, patients with SEMS experience a lower stent failure rate, lower rate of cholangitis, and fewer chemotherapy interruptions than patients with plastic stents.

**Supplementary Information:**

The online version contains supplementary material available at 10.1007/s00464-021-08327-y.

Endoscopic biliary drainage is commonly used to relieve obstructive jaundice in pancreatic cancer (PAC) patients. Obstructive jaundice is associated with poor liver function, impaired coagulation, and the development of cholangitis, which increases morbidity among PAC patients [1]. Cholangitis and high serum bilirubin levels are also contraindications for chemotherapy. Yet, appropriate chemotherapy lengthens life expectancy in patients with inoperable PAC [2]. Furthermore, an overall improvement in survival also emerges in operative neoadjuvant-treated PAC patients [2]. To allow chemotherapy regimens to proceed as planned, managing biliary obstruction and preventing cholangitis remain extremely important.

Both plastic stents and self-expandable metallic stents (SEMSes) have been used for biliary decompression (BD). In recent studies, SEMS has been considered superior to plastic stents, particularly in patients receiving neoadjuvant chemotherapy (NAT) [3–5]. Guidelines recommend using SEMS in NAT patients with PAC [6]. It is thought that the larger diameter of SEMS contributes to its lower rate of preoperative stent complications. Some studies, however, found no difference in post-surgical complication rates when compared to biliary drainage using plastic stents [7]. For patients undergoing pancreatoduodenectomy (PD), preoperative biliary drainage is controversial, with some research indicating no advantage in postoperative complications compared to surgery without preoperative biliary drainage [8, 9]. Previously, biliary stenting was thought to increase the number of postoperative infections, although recent studies have contradicted such results [10, 11]. Furthermore, guidelines and studies do not recommend routine preoperative biliary drainage [6, 9]. However, biliary drainage preoperatively is indicated for patients with cholangitis or symptomatic jaundice with intense pruritus. If surgery is delayed for any reason, or if a jaundiced patient receives NAT, biliary drainage is also indicated [6, 12]. Prolonged obstructive jaundice is a risk for developing cholangitis and other complications possibly later affecting surgery [12].

Stent-related complications are fairly common in patients with advanced PAC receiving palliative chemotherapy (PT) and increase patient morbidity [13]. It is important to understand how these stent-related events can be prevented, and the choice of stent represents an important factor. Guidelines recommend SEMS for palliative biliary drainage in jaundiced patients [6]. However, SEMS might be related to the development of nonocclusion cholangitis in PAC patients with PT [14]. SEMS is not recommended for biliary drainage, if the histopathology of the obstructive process is unclear [6]. Removable fully covered SEMSs could be one option, but they aren’t widely used. Some centers prefer plastic stents, which are exchanged every three months to prevent stent occlusion or the formation of cholangitis. However, studies have shown that a shorter exchange interval should be considered with plastic stents to prevent clogging and cholangitis [15].

Here, we hypothesized that PAC patients receiving chemotherapy experience less cholangitis, fewer repeat endoscopic retrograde cholangiopancreatographies (ERCPs) and fewer chemotherapy interruptions when SEMS are used for BD instead of plastic stents. We aimed to confirm the superiority of SEMS by analyzing the differences in stent complication, cholangitis, and chemotherapy interruption rates in PAC patients receiving plastic stents and SEMSes in our hospital.

## Materials and methods

All patients undergoing BD via ERCP during 2000–2017 at Helsinki University Hospital were identified. Among those patients, we selected those with PAC receiving NAT or PT. Due to the retrospective design of this study and analysis of existing data, this study was conducted without obtaining consent from the participants. For the same reasons permission from our hospital ethics board was not needed, either. ERCPs were performed under conscious sedation by experienced endoscopists, each of whom performs over 300 ERCPs annually. The decision on whether to place a SEMS or PS on the initial ERCP procedure was made according to ESGE guidelines [6]. If the patient had a confirmed malignant disease, (i.e., PAD sample confirming the malignancy, or a metastatic disease was confirmed in CT imaging or MRI), a SEMS was placed. If malignancy could not be confirmed before stent placement, PS was used.The decision on performing a repeat ERCP was made when the stent was thought to be obstructed. The patient was either presenting clinical signs of cholangitis or the patient presented signs of stent failure without cholangitis. We excluded patients undergoing a routine plastic stent exchange without stent complications (*n* = 2).

The stent type (plastic stent or SEMS), procedure date, appearance of cholangitis, the need for stent exchange, the type of chemotherapy received, and the number of chemotherapy interruptions were collected. The plastic stents used were mainly 5–7-cm-long Tannenbaum stents with a diameter of 10 Fr. The SEMSes used were primarily 6–8-cm-long Hanarostents (Olympus Medical) or WallFlex (Boston Scientific) stents expanding to a diameter of 10 mm. All SEMSes were uncovered. All stents used were purchased by our endoscopy unit, and there were no sponsored stents.

Cholangitis was defined through clinical findings. These clinical signs included elevated serum bilirubin levels > 40 umol/l, an elevated body temperature > 38.0 °C and/or an elevated blood serum C-reactive protein (CRP) > 50 mg/l combined with a thickening of the biliary duct walls visible from a CT scan and/or visually infected biliary fluid observed during endoscopy. Stent failure without cholangitis was defined as a patient presenting with a serum bilirubin > 40 umol/l, patient suffering from jaundice, and/or imaging showing no air in the biliary ducts, indicating stent occlusion even if the patient did not show symptoms of an infection. Chemotherapy regimens varied. Among NAT and PT patients, gemcitabine alone or gemcitabine combined with cisplatin or paclitaxel served as the most common treatments administered. Some patients received Folfirinox as the first-line treatment. All patients received at least one cycle of chemotherapy and some patients underwent radiation therapy combined with their treatment (see Supplemental Table 1 for further details). Additional radiation therapy was given to NAT patients if the tumor was adhering to the celiac artery or superior mesenteric artery. In PT patients, additional radiation therapy was given if the disease progressed during chemotherapy. However, all PT patients with disease progression during chemotherapy did not receive radiation therapy. In our material, patients receiving additional radiation therapy were not found to have more stent complications than patients receiving NAT or PT without radiation therapy.

Age, the American Society of Anesthesiologists’ (ASA) physical status classification, and body mass index (BMI) were documented upon primary stent placement. NAT patients were followed from primary biliary drainage until PD or exploratory surgery. PT patients were followed from primary biliary drainage until death. Routine follow-up of plastic stent patients ended if the stent was swapped for a SEMS. See Table 1 additional for details. We also noted any ERCP-related complications. The definition and classification of ERCP-related complications were based on the Cotton consensus criteria [16].

**Table 1:** Patient characteristics

	All, *n* (%)	NAT, *n* (%)	PT, *n* (%)
Patients participating in the study			
Plastic stent	228 (78)	91 (40)	137 (60)
SEMS	64 (22)	15 (23)	49 (77)

	Median	Min	Max
NAT, plastic stents			
Age (in years)	64.8	43.9	80.7
Follow-up time (in days)	163	20	455
ASA physical status classification (I–IV)	3	2	4
BMI (kg/m^2^)	25.3	16.6	47.9
NAT, SEMS			
Age (in years)	64.6	39.5	78.8
Follow-up time (in days)	170	93	361
ASA physical status classification (I–IV)	3	2	3
BMI (kg/m^2^)	25.5	17.1	40.4
PT, plastic stents			
Age (in years)	67.9	44.1	86.3
Follow-up time (in days)	296	11	1324
ASA physical status classification (I–IV)	3	2	4
BMI (kg/m^2^)	24.4	16.8	47.5
PT, SEMS			
Age (in years)	68.0	51.4	93.1
Follow-up time (in days)	377	7	1228
ASA physical status classification (I–IV)	3	2	4
BMI (kg/m^2^)	22.5	16.8	33.4

*SEMS* self-expandable metallic stent, *NAT* neoadjuvant treatment, *PT* palliative treatment, *ASA* American Society of Anesthesiologists’ physical status classification, *BMI* body mass index

### Statistical analysis

Data are reported as the number of cases and as a percentage or as a median value and range. We tested for statistical significance using the Pearson's chi-square test and the Fisher´s exact test, and considered two-sided *p*-values < 0.05 as significant. Some patients experienced multiple stent-related complications leading to one or more repeat ERCPs. Therefore the Prentice, Williams, and Peterson extension for the Cox proportional hazards model was used to determine the overall risk of developing any stent-related complication during the follow-up period. Univariate analyses were used to evaluate risk for repeat ERCP and stent exchange, cholangitis, and stent occlusion without cholangitis. We calculated the 95% confidence intervals (95% CIs) for the hazard ratios (HRs). All statistical analyses were performed using IBM’s SPSS Statistics.

## Results

A total of 292 patients were enrolled (female 128, 44%), a majority of whom had a plastic stent and received PT. The number of SEMS patients undergoing NAT was low (see Table 1).

### Need for repeat ERCP and stent exchange

Patients with SEMSes needed significantly fewer repeat ERCPs compared with plastic stent patients. Combining all patients undergoing oncological treatments, 10 patients with SEMS (16%) and 121 with plastic stents (53%) needed one or more repeat ERCPs (*p* < 0.001). The superiority of SEMS also emerged when PT and NAT patients were analyzed separately.

In the proportional hazards model, the advantage of SEMS was also visible. PT patients with plastic stents exhibited a 3.5 times higher risk for stent failure than SEMS patients (*p* = 0.0001). A similar pattern emerged among NAT patients. Patients with plastic stents exhibited an almost 10 times higher risk for stent complications than patients with SEMS (*p* = 0.013); see Table 2 for details.

**Table 2:** Need for repeat ERCP and stent exchange

	No repeat ERCP, *n* (%)	1 repeat ERCP, *n* (%)	≥ 2 repeat ERCPs, *n* (%)
PT patients (*n* = 186)			
SEMS	39 (80)	7 (14)	3 (6)
Plastic stents	42 (31)	70 (51)	25 (18)
	*p* < 0.001		
NAT patients (*n* = 106)			
SEMS	15 (100)	0	0
Plastic stents	65 (72)	22 (24)	4 (4)
	*p* = 0.020		

	HR	95% CIs	*p* value
Hazard ratios (HRs) for plastic stents vs. SEMSes			
NAT	9.9	1.6–60.5	0.013
PT	3.5	2.3–5.5	0.001

*PT* palliative treatment, *ERCP* endoscopic retrograde cholangiopancreatography, *NAT* neoadjuvant treatment, *SEMS* self-expandable metallic stent, *HR* hazard ratio for plastic stents, *CI* confidence interval

Among 26 NAT patients with a plastic stent dysfunction, 16 (62%) patients received a SEMS during the first repeat ERCP. The decision on changing the stent type from plastic stent to SEMS was made based on guidelines and studies supporting the replacement of plastic stents to SEMSs on the basis of the underlying disease [6, 17]. Median time from primary biliary drainage to first repeat ERCP among NAT patients with plastic stents was 77 days (range 9–263 days). None of the NAT patients with SEMS required a repeat ERCP. In comparison, the median time from primary stent placement until the first stent complication for PT patients with plastic stents was 131 days (range 7–693 days). Among PT patients with SEMS, the median time from primary biliary drainage to first repeat ERCP was 172 days (range 13–1047 days).

Among 95 PT patients with plastic stents requiring repeat ERCP, 76 (80%) received a SEMS during the first repeat ERCP. An additional 5 PT with plastic stents received a percutaneous biliary drain after the first repeat ERCP failed due to duodenum obstruction or some other inability to reach the papilla. All ten patients with occluded SEMS received PT. An additional SEMS, partially or fully inside the first stent, was placed in seven patients. In two patients the existing SEMS was sweeped from material occluding the stent and in one case a PS was placed inside the SEMS.

### Cholangitis

When all patients undergoing oncological treatments were combined, 8 (13%) patients with SEMSes and 73 (32%) patients with plastic stents developed cholangitis (*p* = 0.002). No patients with SEMSes receiving NAT developed cholangitis. One patient suffered from post-ERCP cholecystitis, but that did not affect their chemotherapy nor did the patient require a repeat ERCP. Unfortunately, the number of NAT patients with SEMSes in this study was low. Thus, the Pearson's chi-square test revealed no significant difference in the results between plastic stents and SEMSes. However, our results indicate a slight indication of the superiority of SEMS in NAT patients as well. A proportional hazards model analysis showed that NAT patients with plastic stents exhibited a 4.8 times higher risk for cholangitis than patients with SEMSes (*p* = 0.054). This difference also emerged in PT patients. Patients with plastic stents exhibited a 3.7 times higher risk for cholangitis compared with patients with SEMSes (see Table 3 for details).

**Table 3:** Cholangitis among neoadjuvant treatment (NAT) and palliative treatment (PT) patients with a self-expandable metallic stent (SEMS) or plastic stent

	Cholangitis NAT, *n* (%)	Cholangitis PT, *n* (%)
SEMS	0	8 (16)
Plastic stents	14 (15)	59 (43)
	*p* = 0.211	*p* < 0.001

	HR (95% CIs)	*p* value
Hazard ratios (HRs) for plastic stents vs SEMSes		
NAT	4.8 (0.96–23.7)	*p* = 0.055
Palliative	3.7 (1.6-4.5)	*p* = 0.0001

*NAT* neoadjuvant treatment, *PT* palliative treatment, *SEMS* self-expandable metallic stent, *HR* hazard ratio for plastic stents, *CI* confidence interval

### Stent occlusion or migration without cholangitis

None of the NAT patients with SEMSes experienced stent occlusion problems, whereas 10 NAT patients with plastic stents (11.0%) experienced stent occlusion or migration (*p* > 0.10). Among PT patients, two (4.1%) patients with SEMSes experienced stent occlusion without cholangitis, whereas 29 (20%) patients with plastic stents experienced problems with stent occlusion or migration (*p* = 0.10; see Table 4). An additional 5 patients underwent stent changes due to simultaneous stenting for duodenum obstruction.

**Table 4:** Stent occlusion among neoadjuvant treatment (NAT) and palliative treatment (PT) patients with a self-expandable metallic stent (SEMS) or plastic stent

	Stent occlusion PT, *n* (%)	Stent occlusion NAT, *n* (%)
SEMS	0	2 (3%)
Plastic stents	10 (11%)	29 (21%)
	*p* = 0.10	*p* > 0.10

	HR (95% CIs)	*p* value
Hazard ratio (HR) for plastic stents vs. SEMSes		
Palliative^a^	4.14 (1.3–13.3)	0.017

*NAT* neoadjuvant treatment, *PT* palliative treatment, *SEMS* self-expandable metallic stent, *HR* hazard ratio for plastic stents, *CI* confidence interval

^a^The hazard ratio for plastic stents among NAT patients could not be calculated due to the low number of events

Given the low number of events, we could not calculate the proportional hazards model for the risk of stent occlusion among NAT patients. For PT patients, however, our analysis showed that patients with plastic stents exhibited a more than three times higher risk for stent occlusion or stent migration than patients with SEMSes (*p* = 0.017; Table 4).

### Interruption of chemotherapy

Combining all patients receiving NAT or PT, cholangitis interrupted chemotherapy 6 times (9.4%) among patients with SEMSes and 61 times (27%) among patients with plastic stents (*p* = 0.004). Stent occlusion without cholangitis interrupted NAT or PT twice (2.1%) among patients with SEMSes and 31 times (14%) among patients with plastic stents (*p* = 0.023; see Table 5).

**Table 5:** Chemotherapy interruption due to cholangitis or stent occlusion without cholangitis among all 293 palliative and neoadjuvant patients

	Cholangitis, *n* (%)	Stent occlusion without cholangitis, *n* (%)
SEMS (*n* = 64)	6 (9)	2 (2)
Plastic stents (*n* = 229)	61 (27)	31 (14)
*p* value	0.004	0.023

*SEMS* self-expandable metallic stent

NAT patients with SEMSes did not experience any chemotherapy interruptions prior to surgery, whereas cholangitis interrupted chemotherapy in 10 (11%) NAT patients with plastic stents (*p* = 0.351). Among NAT patients with plastic stent, 2 (2.1%) experienced chemotherapy interruptions due to stent occlusion without cholangitis, while 8 patients required plastic stent replacement due to stent occlusion although their oncological treatment was uninterrupted.

Among SEMS patients with PT, 6 (12%) experienced interruptions to their oncological treatment due to cholangitis and two patients had cholangitis not affecting treatment. In comparison, 51 PT patients (40%) with a plastic stent experienced cholangitis that interrupted chemotherapy (*p* = 0.001).

In one (2.0%) PT patient with SEMS, stent occlusion not accompanied by cholangitis interrupted treatment. In comparison, 13 patients (9.4%) with plastic stents experienced interruptions to their PT due to stent occlusion without cholangitis (*p* = 0.006).

### ERCP complications

We identified no severe complications connected to ERCP. Two patients experienced postprocedural bleeding. In both cases, a new duodenoscopy was completed, and no active bleeding was observed. Both cases of bleeding were conservatively managed. A total of 6 patients experienced post-ERCP pancreatitis (PEP) with mild abdominal pain and serum amylase levels > 330 U/L. All PEP cases were mild and managed conservatively. Perforations or cholangitis due to ERCP could not be identified.

## Discussion

In our study, both PT and NAT PAC patients with SEMSes required fewer repeated ERCPs than patients with plastic stents. These findings agree with recent previous studies [3–5]. SEMS patients also experienced fewer cases of cholangitis and chemotherapy interruptions among patients receiving both PT and NAT. By preventing cholangitis and stent occlusion in NAT patients we can avoid interruption of chemotherapy. With successful chemotherapy we can hopefully push more borderline resectable patients within reach of surgery. The effect of the decreased stent complication rate, cholangitis rate, and chemotherapy interruption rates on life expectancy should be further investigated. In addition, the SEMS used in this study were uncovered. A recent study compared fully covered SEMSes and uncovered SEMSes, suggesting that both stents provide similar preoperative management of biliary obstruction in patients with PAC receiving NAT [18]. A study comparing fully covered and uncovered SEMSes in PT patients would also prove useful.

Some endoscopic centers prefer plastic stents given their lower cost and removability in situations where the histopathology remains unclear. However, if we can avoid any excess endoscopic procedures and prevent patients from developing cholangitis, the total cost per patient would be much lower. Covered metallic stents could serve as one option [16]. In our hospital, the cost for an ERCP procedure with a plastic stent is €1300 versus €1900 for SEMS. Examining all PAC patients undergoing chemotherapy, over 52% of patients with plastic stents required an additional endoscopy due to stent occlusion, whereas this figure fell to slightly more than 15% among SEMS. In addition, the cost of a hospital stay should be considered. If a patient experiences acute obstructive jaundice or cholangitis due to stent occlusion, the median stay in a hospital in our unit is three days. The median cost for one day on our surgical ward is €620. This does not include the cost of imaging, laboratory tests, or surgical or endoscopic procedures. In this study, chemotherapy patients with plastic stents developed cholangitis more than three times more often than patients with SEMSes. Therefore, these differences in relation to the need for a hospital stay and accompanying costs are not insignificant. The use of SEMS thus appears cost efficient. Patients with SEMS develop cholangitis less often, undergo fewer repeat endoscopies due to stent failure, and thus require fewer hospitalization days. A further cost analysis is necessary to strengthen these findings.

The strengths of this study include its large cohort and unambiguous results in agreement with previous studies and guidelines [3–6]. One limitation to this study lies in its retrospective design. Furthermore, the number of NAT patients with SEMSes in our study was quite low. Therefore, we could not adequately statistically analyze the cholangitis rates and chemotherapy interruption rates among NAT patients. However, our findings strongly indicate that patients receiving NAT also benefit from SEMS, in agreement with current guidelines and recommendations [6].

To conclude, our study reinforces the superiority of SEMSes compared to plastic stents. We recommend SEMSes for biliary decompression among patients with PAC undergoing chemotherapy. However, additional considerations should be included in treatment situations where the histopathology of the obstruction process remains unclear. Fully covered SEMSes may be considered. If a plastic stent is placed due to an unclear histopathology, it should be swapped out for a SEMS if a stent complication occurs as soon as the obstructive process is confirmed as malignant. In both PT and NAT patients, SEMS associate with a lower stent failure rate, a lower rate of cholangitis, and fewer chemotherapy interruptions than plastic stents.

## Supplementary Information

Below is the link to the electronic supplementary material.

Supplementary Information 1 (DOCX 301 kb)
