# Supplementary material for: Use of self-expandable metallic stents for endoscopic biliary decompression decreases stent complications in pancreatic cancer patients receiving chemotherapy
Source: Surg Endosc. 2021 Feb 3;36(1):614–20. doi: 10.1007/s00464-021-08327-y (PMC8741707; doi:10.1007/s00464-021-08327-y)
Supplement: Supplementary file 1 — Supplementary Information 1 (DOCX 301 kb) [file 464_2021_8327_MOESM1_ESM.docx]

Supplemental table 1. Chemotherapy and radiotherapy regimens patients received. Some patients received chemotherapy in other domestic and foreign hospitals and, therefore, the information regarding the chemotherapy received is unknown. Abbreviation: SEMS, self-expandable metallic stent.

|  | **Chemotherapy** | **Radiotherapy** |
| --- | --- | --- |
|  |  |  |
|  |  |  |
| ***Neoadjuvant treatment, plastic stent*** |  |  |
|  | Gemcitabine, 8 cycles. |  |
|  | Gemcitabine, 1 cycle. |  |
|  | Gemcitabine + Erlotinib, 3 cycles. |  |
|  | Gemcitabine, 6 cycles. | Yes |
|  | Gemcitabine + Paclitaxel, 3 cycles. |  |
|  | Gemcitabine + Cisplatin, 7 cycles. |  |
|  | Gemcitabine + Cisplatin, 3 cycles. |  |
|  | Gemcitabine + Cisplatin, 3 cycles. | Yes |
|  | Gemcitabine + Cisplatin, 4 cycles. |  |
|  | Gemcitabine, 3 cycles. |  |
|  | Folfirinox, 5 cycles. | Yes |
|  | Gemcitabine, 4 cycles. | Yes |
|  | Gemcitabine, 3 cycles. |  |
|  | Gemcitabine, 5 cycles. |  |
|  | Gemcitabine + Cisplatin, 5 cycles. |  |
|  | Gemcitabine + Cisplatin, 3 cycles. | Yes |
|  | Gemcitabine, 3 cycles. |  |
|  | Gemcitabine + Cisplatin, 3 cycles. |  |
|  | Folfirinox, 4 cycles. |  |
|  | Gemcitabine + Cisplatin, 3 cycles. |  |
|  | Gemcitabine + Cisplatin, 4 cycles. |  |
|  | Gemcitabine, 4 cycles. |  |
|  | Gemcitabine, 8 cycles. |  |
|  | Gemcitabine + Cisplatin, 4 cycles. |  |
|  | Folfirinox, 9 cycles. |  |
|  | Carboplatin + Paclitaxel, 3 cycles. | Yes |
|  | Gemcitabine + Cisplatin, 4 cycles. | Yes |
|  | Gemcitabine, 3 cycles. |  |
|  | Unknown. |  |
|  | Folfirinox, 3 cycles. |  |
|  | Gemcitabine + Cisplatin, 4 cycles. | Yes |
|  | Folfirinox, 7 cycles. |  |
|  | Gemcitabine + Paclitaxel, 2 cycles. |  |
|  | Gemcitabine + Cisplatin, 4 cycles. |  |
|  | Paclitaxel, 5 cycles. |  |
|  | Gemcitabine + Cisplatin, 4 cycles. |  |
|  | Unknown. | Yes |
|  | Gemcitabine + Paclitaxel, 3 cycles. |  |
|  | Folfirinox, 4 cycles. Gemcitabine, 3 cycles. |  |
|  | Gemcitabine + Cisplatin, 3 cycles |  |
|  | Unknown. | Yes |
|  | Gemcitabine + Paclitaxel, 3 cycles. |  |
|  | Gemcitabine + Cisplatine, 3 cycles. | Yes |
|  | Unknown. |  |
|  | Gemcitabine + Cisplatine, 3 cycles. |  |
|  | Gemcitabine + Cisplatine, 3 cycles. |  |
|  | Unknown. | Yes |
|  | Gemcitabine + Paclitaxel, 3 cycles. |  |
|  | Gemcitabine + Paclitaxel, 3 cycles. |  |
|  | Unknown. | Yes |
|  | Gemcitabine + Cisplatin, 3 cycles. | Yes |
|  | Gemcitabine + Cisplatin, 3 cycles. |  |
|  | Gemcitabine + Cisplatin, 3 cycles. |  |
|  | Folfirinox, 6 cycles. |  |
|  | Unknown. | Yes |
|  | Gemcitabine, 3 cycles. |  |
|  | Gemcitabine + Cisplatin, 3 cycles. |  |
|  | Gemcitabine, 3 cycles. | Yes |
|  | Folfirinox, 6 cycles. |  |
|  | Gemcitabine + Cisplatin, 3 cycles. |  |
|  | Unknown. | Yes |
|  | Unknown. | Yes |
|  | Gemcitabine, 3 cycles. |  |
|  | Gemcitabine + Paclitaxel, 2 cycles. |  |
|  | Unknown. | Yes |
|  | Gemcitabine + Cisplatin, 4 cycles |  |
|  | Gemcitabine + Cisplatin, 5 cycles. |  |
|  | Gemcitabine, 3 cycles. | Yes |
|  | Unknown. | Yes |
|  | Gemcitabine + Paclitaxel, 4 cycles. |  |
|  | Gemcitabine, 3 cycles. |  |
|  | Folfirinox, 5 cycles. |  |
|  | Unknown. | Yes |
|  | Gemcitabine, 3 cycles. | Yes |
|  | Gemcitabine + Cisplatin, 3 cycles. |  |
|  | Gemcitabine + Cisplatin, 3 cycles. |  |
|  | Folfirinox, 9 cycles. |  |
|  | Unknown. | Yes |
|  | Gemcitabine + Cisplatin, 4 cycles. |  |
|  | Gemcitabine + Cisplatin, 6 cycles. |  |
|  | Gemcitabine, 2 cycles. |  |
|  | Gemcitabine, 3 cycles. |  |
|  | Unknown. | Yes |
|  | Carboplatin + Etoposide, 3 cycles. |  |
|  | Gemcitabine + Cisplatin, 3 cycles. |  |
|  | Gemcitabine, 3 cycles. |  |
|  | Gemcitabine + Cisplatin, 4 cycles. |  |
|  | Gemcitabine + Cisplatin, 3 cycles. |  |
|  | Unknown. | Yes |
|  | Unknown. |  |
|  | Gemcitabine + Cisplatin, 3 cycles. |  |
|  | **Chemotherapy** | **Radiotherapy** |
| ***Neoadjuvant treatment, SEMS*** |  |  |
|  | Gemcitabine + Cisplatin, 3 cycles. | Yes |
|  | Gemcitabine + Cisplatin, 3 cycles. |  |
|  | Folfirinox, 2 cycles. Gemcitabine + Oxaliplatin, 3 cycles. | Yes |
|  | Unknown. |  |
|  | Gemcitabine + Paclitaxel, 2 cycles. |  |
|  | Gemcitabine, 9 cycles. |  |
|  | Gemcitabine, 3 cycles. |  |
|  | Gemcitabine + Cisplatin, 3 cycles. |  |
|  | Gemcitabine + Cisplatin, 9 cycles. |  |
|  | Gemcitabine + Cisplatin, 3 cycles. | Yes |
|  | Gemcitabine, 1 cycle. | Yes |
|  | Gemcitabine + Cisplatin, 3 cycles. |  |
|  | Gemcitabine + Cisplatin, 3 cycles. |  |
|  | Gemcitabine + Cisplatin, 4 cycles. |  |
|  | Gemcitabine, 3 cycles. | Yes |
|  | **Chemotherapy** | **Radiotherapy** |
| ***Palliative treatment, plastic stent*** |  |  |
|  | Gemcitabine + Cisplatin, 3 cycles. |  |
|  | Gemcitabine + Paclitaxel, 8 cycles. Oxaliplatin + Capecitabine, 5 cycles. |  |
|  | Gemcitabine, 7 cycles. |  |
|  | Gemcitabine, 6 cycles. Irinotecan + Capesitabine, 2 cycles. |  |
|  | Gemcitabine + Cisplatin, 6 cycles. Oxaliplatin + Capecitabine, 6 cycles. | Yes |
|  | Gemcitabine + Paclitaxel, 5 cycles. Oxaliplatin + Capecitabine, 8 cycles. |  |
|  | Erlotinib for 2 months. |  |
|  | Folfirinox, 12 cycles. Gemcitabine + Paclitaxel, 5 cycles. |  |
|  | Gemcitabine + Paclitaxel, 7 cycles. Capecitabine, 5 cycles. |  |
|  | Gemcitabine + Cisplatin, 11 cycles. Oxaliplatin + Capecitabine, 2 cycles. |  |
|  | Gemcitabine + Cisplatin, 5 cycles. Capecitabine + Erlotinib, 1 cycle. |  |
|  | Gemcitabine + Paclitaxel, 1 cycle. |  |
|  | Gemcitabine + Cisplatin, 15 cycles. |  |
|  | Gemcitabine + Cisplatin, 7 cycles. Gemcitabine + Capecitabine, 1 cycle. | Yes |
|  | Gemcitabine + Cisplatin, 4 cycles. |  |
|  | Gemcitabine + Cisplatin, 8 cycles. Gemcitabine + Erlotinib, 3 cycles. |  |
|  | Gemcitabine, 10 cycles. Capecitabine, 2 cycles. |  |
|  | Gemcitabine, 3 cycles. |  |
|  | Gemcitabine, 2 cycles. Oxaliplatin + Capecitabine, 4 cycles. | Yes |
|  | Gemcitabine + Paclitaxel, 1 cycle. |  |
|  | Gemcitabine, 2 cycles. | Yes |
|  | Gemcitabine, 9 cycles. Capecitabine + Erlotinib, 2 cycles. |  |
|  | Gemcitabine + Paclitaxel, 4 cycles. |  |
|  | Gemcitabine, 14 cycles. Oxaliplatin + Capecitabine, 3 cycles. |  |
|  | Gemcitabine + Paclitaxel, 4 cycles. Gemcitabine, 3 cycles. Gemcitabine + Capecitabine, 3 cycles. Irinotecan + Capesitabine, 7 cycles. Oxaliplatin + Capecitabine, 3 cycles. |  |
|  | Gemcitabine, 10 cycles. Oxaliplatin + Capecitabine, 3 cycles. |  |
|  | Carboplatin + Etoposide, 2 cycles. |  |
|  | Gemcitabine, 1 cycle. |  |
|  | Gemcitabine + Cisplatin, 3 cycles. | Yes |
|  | Gemcitabine, 1 cycle. |  |
|  | Folfirinox, 1 cycle. Gemcitabine + Cisplatin, 3 cycles. |  |
|  | Gemcitabine, 2 cycles. Gemcitabine + Paclitaxel, 2 cycles. Capecitabine, 1 cycle. |  |
|  | Gemcitabine, 1 cycle. |  |
|  | Gemcitabine, 1 cycle. |  |
|  | Gemcitabine, 2 cycles. |  |
|  | Gemcitabine + Paclitaxel, 2 cycles. Gemcitabine, 1 cycle. | Yes |
|  | Gemcitabine + Cisplatin, 3 cycles. Gemcitabine, 2 cycles. |  |
|  | Gemcitabine, 2 cycles. |  |
|  | Gemcitabine + Cisplatin, 3 cycles. |  |
|  | Folfirinox, 10 cycles. Gemcitabine + Capecitabine, 8 cycles. Capecitabine + Erlotinib for 2 months. |  |
|  | Gemcitabine, 1 cycle. |  |
|  | Gemcitabine + Cisplatin, 2 cycles. Gemcitabine, 2 cycles. |  |
|  | Gemcitabine, 1 cycle. |  |
|  | Gemcitabine, 3 cycles. |  |
|  | Gemcitabine, 1 cycle. |  |
|  | Gemcitabine, 2 cycles. |  |
|  | Gemcitabine + Cisplatin, 6 cycles. Oxaliplatin + Capecitabine, 3 cycles. |  |
|  | Folfirinox, 4 cycles. Gemcitabine, 1 cycle. Gemcitabine + Paclitaxel, 2 cycles. |  |
|  | Gemcitabine + Cisplatin, 1 cycle. Gemcitabine, 2 cycles. |  |
|  | Gemcitabine + Cisplatin, 4 cycles. Oxaliplatin + Capecitabine, 3 cycles. |  |
|  | Gemcitabine, 4 cycles. |  |
|  | Gemcitabine, 1 cycle. |  |
|  | Gemcitabine + Paclitaxel, 2 cycles. |  |
|  | Gemcitabine + Paclitaxel, 5 cycles. Oxaliplatin + Capecitabine, 2 cycles. |  |
|  | Gemcitabine + Cisplatin, 3 cycles. |  |
|  | Gemcitabine, 3 cycles. |  |
|  | Gemcitabine + Cisplatin, 3 cycles. Oxaliplatin + Capecitabine, 1 cycle. |  |
|  | Gemcitabine, 2 cycles. |  |
|  | Gemcitabine, 1 cycle. |  |
|  | Gemcitabine + Cisplatin, 2 cycles. |  |
|  | Gemcitabine + Cisplatin, 6 cycles. Gemcitabine, 1 cycle. Oxaliplatin + Capecitabine, 2 cycles. | Yes |
|  | Gemcitabine + Paclitaxel, 6 cycles. | Yes |
|  | Gemcitabine + Cisplatin, 3 cycles. |  |
|  | Gemcitabine + Paclitaxel, 10 cycles. Oxaliplatin + Capecitabine, 5 cycles. |  |
|  | Gemcitabine, 1 cycle. |  |
|  | Gemcitabine, 3 cycles. |  |
|  | Gemcitabine + Cisplatin, 3 cycles. Gemcitabine + Capecitabine, 6 cycles. Irinotecan + Capesitabine, 2 cycles. | Yes |
|  | Gemcitabine, 3 cycles. |  |
|  | Gemcitabine, 1 cycle. |  |
|  | Folfirinox, 5 cycles. Gemcitabine, 3 cycle. | Yes |
|  | Gemcitabine, 1 cycle. Gemcitabine + Capecitabine, 2 cycles. |  |
|  | Gemcitabine, 6 cycles. |  |
|  | Gemcitabine + Cisplatin, 3 cycles. Irinotecan + Capesitabine, 1 cycle. |  |
|  | Gemcitabine + Paclitaxel, 2 cycles. |  |
|  | Gemcitabine, 3 cycles. |  |
|  | Gemcitabine + Paclitaxel, 8 cycles. |  |
|  | Gemcitabine, 3 cycles. Oxaliplatin + Capecitabine, 7 cycles. | Yes |
|  | Gemcitabine, 1 cycle. |  |
|  | Gemcitabine + Paclitaxel, 8 cycles. |  |
|  | Gemcitabine, 4 cycles. |  |
|  | Folfirinox, 9 cycles. Gemcitabine + Paclitaxel, 2 cycles. | Yes |
|  | Gemcitabine, 3 cycles. |  |
|  | Gemcitabine + Cisplatin, 3 cycles. Gemcitabine, 3 cycles. |  |
|  | Gemcitabine + Cisplatin, 6 cycles. |  |
|  | Gemcitabine, 2 cycles. |  |
|  | Gemcitabine, 5 cycles. |  |
|  | Gemcitabine, 4 cycles. |  |
|  | Folfirinox, 7 cycles. |  |
|  | Folfirinox, 6 cycles. Gemcitabine + Cisplatin, 11 cycles. Irinotecan + Capesitabine, 1 cycle. |  |
|  | Folfirinox, 10 cycles. |  |
|  | Gemcitabine, 6 cycles. Capecitabine, 2 cycles. |  |
|  | Gemcitabine, 1 cycle. Gemcitabine + Paclitaxel, 2 cycles. |  |
|  | Gemcitabine + Capecitabine, 2 cycles. Gemcitabine, 1 cycle. Irinotecan + Capesitabine, 2 cycles. |  |
|  | Gemcitabine + Cisplatin, 11 cycles. Gemcitabine, 3 cycles. Irinotecan + Capesitabine, 4 cycles. |  |
|  | Gemcitabine, 3 cycles. Oxaliplatin + Capecitabine, 3 cycles. |  |
|  | Gemcitabine + Cisplatin, 1 cycle. |  |
|  | Gemcitabine, 14 cycles. Oxaliplatin + Capecitabine, 2 cycles. |  |
|  | Gemcitabine + Paclitaxel, 6 cycles. Irinotecan + Capesitabine, 1 cycle. |  |
|  | Gemcitabine, 6 cycles. |  |
|  | Gemcitabine, 5 cycles. |  |
|  | Gemcitabine + Paclitaxel, 4 cycles | Yes |
|  | Gemcitabine + Paclitaxel, 7 cycles | Yes |
|  | Gemcitabine, 8 cycles. |  |
|  | Gemcitabine, 6 cycles. |  |
|  | Gemcitabine, 4 cycles. |  |
|  | Gemcitabine, 3 cycles. | Yes |
|  | Gemcitabine, 1 cycle. Capecitabine + Oxaliplatin, 1 cycle. |  |
|  | Gemcitabine + Cisplatin, 5 cycles. |  |
|  | Gemcitabine, 3 cycles. Capecitabine + Oxaliplatin, 2 cycles. |  |
|  | Gemcitabine + Cisplatin, 9 cycles. |  |
|  | Gemcitabine + Cisplatin, 3 cycles. Gemcitabine, 6 cycles, |  |
|  | Gemcitabine + Cisplatin, 5 cycles. Gemcitabine, 3 cycles, Capecitabine + Oxaliplatin, 2 cycles. |  |
|  | Gemcitabinr-Paclitaxel, 10 cycles. Irinotecan + Capesitabine, 3 cycles. |  |
|  | Folfirinox, 2 cycles. |  |
|  | Gemcitabine, 3 cycles. Capecitabine + Oxaliplatin, 1 cycle. |  |
|  | Gemcitabine, 1 cycle. |  |
|  | Gemcitabine + Cisplatin, 1 cycle. Gemcitabine, 2 cycles. |  |
|  | Gemcitabine + Cisplatin, 15 cycles. Gemcitabine + Capecitabine , 1 cycle. |  |
|  | Gemcitabine + Cisplatin, 3 cycles. |  |
|  | Gemcitabine, 8 cycles. Capecitabine + Oxaliplatin, 3 cycles. |  |
|  | Gemcitabine + Cisplatin, 3 cycles. Capecitabine + Oxaliplatin, 8 cycles. |  |
|  | Gemcitabine + Paclitaxel, 3 cycles. Gemcitabine, 1 cycle. Gemcitabine + Capecitabine, 3 cycles. | Yes |
|  | Gemcitabine, 9 cycles. Capecitabine + Oxaliplatin, 8 cycles. |  |
|  | Gemcitabine + Paclitaxel, 7 cycles. | Yes |
|  | Folfirinox, 3 cycles. Gemcitabine + Paclitaxel, 11 cycles. |  |
|  | Gemcitabine + Cisplatin, 9 cycles. Gemcitabine + Capecitabine, 3 cycles. |  |
|  | Gemcitabine, 6 cycles. |  |
|  | Gemcitabine + Paclitaxel, 11 cycles. |  |
|  | Gemcitabine, 14 cycles. |  |
|  | Gemcitabine + Cisplatin, 6 cycles. | Yes |
|  | Folfirinox, 1 cycle. Gemcitabine + Cisplatin, 6 cycles. Gemcitabine + Erlotinib, 11 cycles. Capesitabine + Oxaliplatin, 2 cycles. Irinotecan + Capesitabine, 2 cycles. |  |
|  | Gemcitabine + Capecitabine, 7 cycles. |  |
|  | Gemcitabine + Paclitaxel, 11 cycles. |  |
|  | Gemcitabine + Paclitaxel, 27 cycles. Irinotecan + Capesitabine, 7 cycles. |  |
|  | Gemcitabine + Paclitaxel, 3 cycles. |  |
|  | Gemcitabine, 8 cycles. |  |
|  | Gemcitabine, 37 cycles. |  |
|  | **Chemotherapy** | **Radiotherapy** |
| ***Palliative treatment, SEMS*** |  |  |
|  | Gemcitabine, 1 cycle. |  |
|  | Gemcitabine + Cisplatin, 6 cycles. |  |
|  | Gemcitabine + Cisplatin, 1 cycle. |  |
|  | Gemcitabine + Cisplatin, 4 cycles. Gemcitabine, 4 cycles. | Yes |
|  | Gemcitabine, 3 cycles. Oxaliplatin + Capecitabine, 3 cycles, Irinotecan + Capesitabine, 6 cycles. | Yes |
|  | Gemcitabine, 2 cycles. |  |
|  | Folfirinox, 8 cycles. Gemcitabine + Cabesitabine, 1 cycle. | Yes |
|  | Gemcitabine, 3 cycles. |  |
|  | Gemcitabine + Cisplatin, 2 cycles. Gemcitabine, 6 cycles. |  |
|  | Gemcitabine, 1 cycle. |  |
|  | Gemcitabine + Cisplatin, 8 cycles. |  |
|  | Gemcitabine, 19 cycles. Oxaliplatin + Capecitabine, 3 cycles. |  |
|  | Gemcitabine, 2 cycles. |  |
|  | Gemcitabine, 3 cycles. Oxaliplatin + Capecitabine, 3 cycles. |  |
|  | Gemcitabine, 6 cycles. Cabecitabine, 2 cycles. |  |
|  | Gemcitabine + Paclitaxel, 5 cycles. | Yes |
|  | Gemcitabine, 1 cycle. |  |
|  | Gemcitabine + Cisplatin, 3 cycles. |  |
|  | Gemcitabine + Cisplatin, 5 cycles. Oxaliplatin + Capecitabine, 3 cycles. | Yes |
|  | Gemcitabine, 23 cycles. Oxaliplatin + Capecitabine, 8 cycles. Gemcitabine + Erlotinib, 2 cycles |  |
|  | Gemcitabine + Cisplatin, 3 cycles. Gemcitabine, 3 cycles. |  |
|  | Gemcitabine + Cisplatin, 3 cycles. |  |
|  | Gemcitabine + Cisplatin, 6 cycles. Gemcitabine, 5 cycles. |  |
|  | Gemcitabine + Paclitaxel, 8 cycles. | Yes |
|  | Gemcitabine, 7 cycles. Oxaliplatin + Capecitabine, 9 cycles, Irinotecan + Capesitabine, 8 cycles. |  |
|  | Gemcitabine, 4 cycles. |  |
|  | Gemcitabine + Cisplatin, 6 cycles. Capecitabine + Erlotinib, 6 cycles. |  |
|  | Gemcitabine + Erlotinib, 6 cycles. |  |
|  | Gemcitabine + Cisplatin, 1 cycle. Gemcitabine, 2 cycles. | Yes |
|  | Gemcitabine, 4 cycles. |  |
|  | Gemcitabine + Cisplatin, 3 cycles. Oxaliplatin + Capecitabine, 1 cycle. | Yes |
|  | Gemcitabine + Cisplatin, 3 cycles. Gemcitabine, 3 cycles. |  |
|  | Gemcitabine + Cisplatin, 6 cycles. Oxaliplatin + Capecitabine, 4 cycles. |  |
|  | Gemcitabine, 2 cycles. |  |
|  | Gemcitabine, 4 cycles. |  |
|  | Gemcitabine + Cisplatin, 6 cycles. Oxaliplatin + Capecitabine, 2 cycles. |  |
|  | Gemcitabine + Cisplatin, 3 cycles. |  |
|  | Folfirinox, 3 cycles. Gemcitabine + Paclitaxel, 3 cycles. Oxaliplatin + Capecitabine, 7 cycles. |  |
|  | Gemcitabine, 6 cycles. |  |
|  | Gemcitabine, 4 cycles. Oxaliplatin + Capecitabine, 3 cycles. |  |
|  | Gemcitabine + Cisplatin, 3 cycles. Gemcitabine, 1 cycle. |  |
|  | Gemcitabine + Cisplatin, 3 cycles. |  |
|  | Gemcitabine, 3 cycles. Folfirinox, 3 cycles. |  |
|  | Gemcitabine, 12 cycles. |  |
|  | Gemcitabine + Paclitaxel, 4 cycles. Oxaliplatin + Capecitabine, 7 cycles. |  |
|  | Gemcitabine, 6 cycles. |  |
|  | Folfirinox, 6 cycles. | Yes |
|  | Gemcitabine, 12 cycles. Capecitabine, 8 cycles. |  |
|  | Gemcitabine, 28 cycles. Oxaliplatin + Capecitabine, 10 cycles. |  |
